# Transient Polycomb activity represses developmental genes in growing oocytes

**DOI:** 10.1186/s13148-022-01400-w

**Published:** 2022-12-21

**Authors:** Ellen G. Jarred, Zhipeng Qu, Tesha Tsai, Ruby Oberin, Sigrid Petautschnig, Heidi Bildsoe, Stephen Pederson, Qing-hua Zhang, Jessica M. Stringer, John Carroll, David K. Gardner, Maarten Van den Buuse, Natalie A. Sims, William T. Gibson, David L. Adelson, Patrick S. Western

**Affiliations:** 1 Centre for Reproductive Health, Hudson Institute of Medical Research, Clayton, VIC Australia; 2 Department of Molecular and Translational Science, Monash University, Clayton, VIC Australia; 3 Department of Molecular and Biomedical Science, School of Biological Sciences, University of Adelaide, Adelaide, SA Australia; 4 Biomedicine Discovery Institute, Monash University, Clayton, VIC Australia; 5 School of BioSciences, University of Melbourne, Parkville, VIC Australia; 6 School of Psychology and Public Health, La Trobe University, Melbourne, VIC Australia; 7 Bone Cell Biology and Disease Unit, St. Vincent’s Institute of Medical Research, Fitzroy, VIC Australia; 8 Department of Medicine at St, Vincent’s Hospital, Fitzroy, VIC Australia; 9 Department of Medical Genetics, University of British Columbia and British Columbia Children’s Hospital Research Institute, Vancouver, BC Canada; 10 South Australian Museum, SA Adelaide, Australia

**Keywords:** Polycomb, Oocyte, Programming, Epigenetic, H3K27me3, Inheritance

## Abstract

**Background:**

Non-genetic disease inheritance and offspring phenotype are substantially influenced by germline epigenetic programming, including genomic imprinting. Loss of Polycomb Repressive Complex 2 (PRC2) function in oocytes causes non-genetically inherited effects on offspring, including embryonic growth restriction followed by post-natal offspring overgrowth. While PRC2-dependent non-canonical imprinting is likely to contribute, less is known about germline epigenetic programming of non-imprinted genes during oocyte growth. In addition, de novo germline mutations in genes encoding PRC2 lead to overgrowth syndromes in human patients, but the extent to which PRC2 activity is conserved in human oocytes is poorly understood.

**Results:**

In this study, we identify a discrete period of early oocyte growth during which PRC2 is expressed in mouse growing oocytes. Deletion of *Eed* during this window led to the de-repression of 343 genes. A high proportion of these were developmental regulators, and the vast majority were not imprinted genes. Many of the de-repressed genes were also marked by the PRC2-dependent epigenetic modification histone 3 lysine 27 trimethylation (H3K27me3) in primary–secondary mouse oocytes, at a time concurrent with PRC2 expression. In addition, we found H3K27me3 was also enriched on many of these genes by the germinal vesicle (GV) stage in human oocytes, strongly indicating that this PRC2 function is conserved in the human germline. However, while the 343 genes were de-repressed in mouse oocytes lacking EED, they were not de-repressed in pre-implantation embryos and lost H3K27me3 during pre-implantation development. This implies that H3K27me3 is a transient feature that represses a wide range of genes in oocytes.

**Conclusions:**

Together, these data indicate that EED has spatially and temporally distinct functions in the female germline to repress a wide range of developmentally important genes and that this activity is conserved in the mouse and human germlines.

**Supplementary Information:**

The online version contains supplementary material available at 10.1186/s13148-022-01400-w.

## Background

Epigenetic modifications, including DNA methylation and histone modifications, regulate chromatin packaging and underlie long-term cell-specific gene transcription patterns. Amongst other chromatin regulatory functions, many of these modifications are essential for cell differentiation and provide mechanisms for maintaining lineage-specific identity and cell functions throughout the life of an organism. Conversely, dysregulation of epigenetic modifications contributes to a wide range of diseases and syndromes, including congenital anomalies, cancer, diabetes and behavioural conditions [1–4].

The maternal and paternal genomes transmit genetic and epigenetic information to offspring at fertilisation. While oocyte and sperm chromatin are respectively organised in distinct histone and protamine-mediated structures, the vast majority of maternal and paternal alleles achieve epigenetic equivalence within a short period after fertilisation, a process that relies partly on proteins and RNAs that are maternally inherited in the oocyte. However, some genes maintain parent-specific epigenetic patterns that were established during sperm and oocyte development. In mice and humans, these genes include around 120 imprinted genes that are typically marked either by maternal or paternal DNA methylation, an epigenetic state that is transmitted to, and maintained in offspring and is essential for parent-of-origin specific gene regulation during development [5–8]. While genomic imprinting provides an unequivocal example of epigenetic inheritance, evidence for other epigenetically inherited states that may affect biallelically expressed genes is rare and the mechanisms underlying such inheritance are poorly understood [5]. Given the potential for epigenetic states to influence offspring development, identifying the specific chromatin-modifying complexes that epigenetically regulate developmental genes and may influence the establishment of an appropriate epigenetic landscape in oocytes would enhance understanding of the mechanisms underlying inherited phenotypes and disease, and of how these mechanisms may contribute to evolution.

Histone 3 lysine 27 trimethylation (H3K27me3) is a critical epigenetic modification catalysed by the Polycomb Repressive Complex 2 (PRC2). PRC2 contains three essential core protein subunits: Suppressor of Zeste 12 (SUZ12), Embryonic Ectoderm Development (EED) and Enhancer of Zeste 1/2 (EZH1/2), all of which are required for histone methyltransferase activity [9–12]. While EZH2 can function in PRC2-independent roles, EED is only known to mediate the methylation of H3K27 as an essential component of PRC2 [13–19]. Specific examples include an essential role for EED in repressing a wide range of developmentally important genes in embryonic stem cells (ESCs) through its essential role in establishing H3K27me3 [12, 20]. While EZH2 also plays a major role in the repression of the same genes, the closely related protein EZH1 acts in a partially redundant manner and contributes both to H3K27me3 enrichment and gene repression [12]. In other contexts, EZH2 can directly methylate non-histone target proteins such as PLZF in B lymphocytes of the immune system, and GATA4 in mouse foetal cardiomyocytes in vivo [13, 17].

PRC2 also plays important roles in sperm and oocytes, and throughout development. De novo germline mutations in human *EED, EZH2* and *SUZ12* underlie Cohen-Gibson, Weaver and Imagawa-Matsumoto syndromes which are characterised by perinatal overgrowth, skeletal malformation and cognitive deficit [21–30]. Multiple studies in mice indicate that EZH2 and EED act as maternal factor proteins and/or mRNA that is required in mature oocytes to regulate the establishment and maintenance of X-inactivation in pre-implantation embryos [31–34]. In addition, PRC2 regulates DNA methylation-independent non-canonical imprinting in mouse oocytes, a process that involves H3K27me3-dependent programming and paternally biased expression of up to 20 genes in pre-implantation embryos and five genes in extraembryonic ectoderm and placenta until embryonic day (E)9.5 [8, 35]. Maternal deletion of *Eed* resulted in the loss of H3K27me3 imprints, biallelic expression of H3K27me3-imprinted genes in pre-implantation embryos and extraembryonic ectoderm, transient ectopic X-inactivation, and male-biased embryo loss [33, 34]. Moreover, mouse offspring generated by somatic cell nuclear transfer (SCNT) are typically born large as a result of placental hyperplasia, a phenotype that is caused by loss of H3K27me3 imprinting primarily of *Slc38a4* and *Sfmbt2-*embedded micro-RNAs specifically in the placenta [36–38]. Although H3K27me3 imprinting specifically affects the placenta, embryonic growth restriction was also observed in embryos derived from oocytes lacking EED, but the cause of this phenotype is not understood [33]. While H3K27me3-dependent imprinting (non-canonical) has been recently identified, classical (or canonical) genomic imprinting is much more extensively studied and is generally considered to be mediated by DNA methylation [8, 39]. Here, we refer to canonical DNA methylation-based genomic imprints as classical imprinting and non-canonical imprinting as H3K27me3-dependent imprinting.

We previously found that deletion of *Eed* in growing oocytes led to post-natal overgrowth of offspring, indicating that maternally derived PRC2 mediates effects on offspring that were independent of maternal genetic inheritance [40]. To understand the potential mechanisms underlying developmental outcomes in offspring from *Eed*-null oocytes, we explored the role of PRC2 in oocytes. We demonstrate that EZH2, EED and SUZ12 are transiently expressed during the earliest stages of oocyte growth to establish H3K27me3 in the promoters of developmentally important genes in mice and that H3K27me3 is conserved on many of these genes in human GV stage oocytes. In mice, PRC2 activity immediately preceded the upregulation of the essential de novo DNA methylation co-factor DNMT3L, indicating that patterning of PRC2 target genes precedes DNA methylation. While *Eed* repressed several imprinted genes in oocytes, 98% of the PRC2 target genes we identified were not imprinted, but were genes that regulate neurogenesis, haematopoiesis and other processes in tissue morphogenesis. These genes were not dysregulated in pre-implantation offspring and lost H3K27me3 during this period of development in wild-type (*wt*) embryos.

## Results

### EZH2, EED and SUZ12 localise to chromatin during a discrete period of primary to secondary oocyte growth

**Fig. 1:**
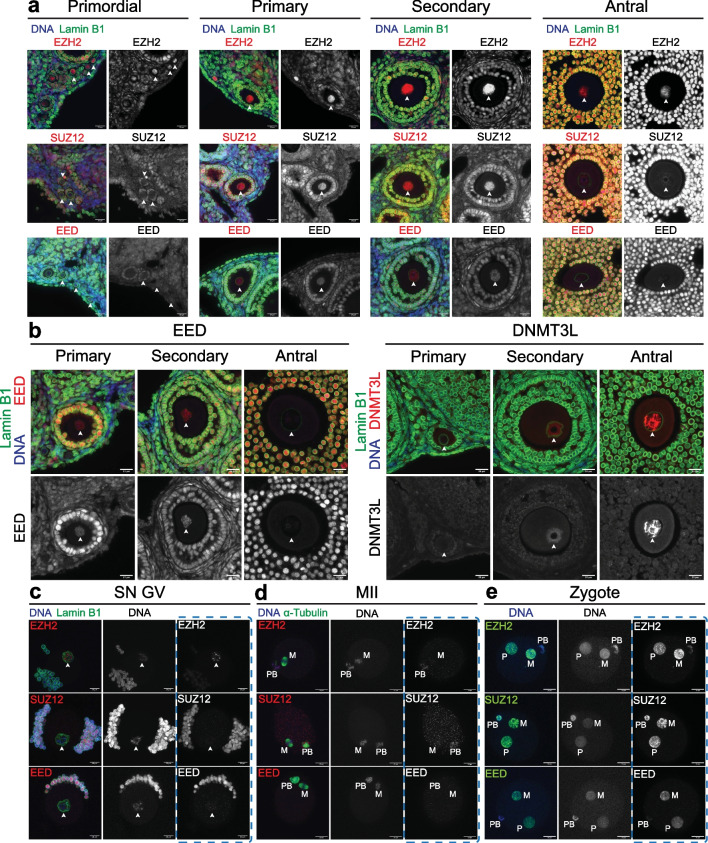
PRC2 acts transiently within primary and secondary follicle growing oocytes. **(a-d)** Representative images of EZH2, SUZ12 and EED (red) IF analysis in **(a)** primordial, primary, secondary and antral follicles. **(b)** Comparison of EED (red, left panels) versus DNMT3L (red, right panels) IF analysis in primary, secondary and antral follicles. **(c)** Surrounded nucleolus (SN) GV oocytes. **(d)** MII oocytes. α-Tubulin (green) identifies meiotic spindles. M: metaphase plate, PB: polar body. **(e)** Zygotes 12 h after fertilisation for ≥ 10 zygotes imaged per antibody combination. M: maternal pronucleus, P: paternal pronucleus, PB: polar body. In **a-c** white arrowheads indicate the oocyte nucleus defined by Lamin B1 (green) and DAPI (blue) shows DNA. In **c-e** images represent compressed z-stack images of whole-mount oocytes or zygotes. Scale bars: 20 μm. Images in** a** and **b** are representative of two ovaries from three separate females and in **c-e** images are representative of ≥ 10 oocytes per antibody combination

Previous studies have provided varying reports of EED, EZH2 and SUZ12 in GV stage and mature oocytes, and zygotes [31, 41–43], but the stages at which all three core components of PRC2 are detected in growing oocytes have not been defined. To determine when PRC2 is detected in the nucleus or associated with chromosomes in growing, GV and MII oocytes, and in zygotes, we profiled EZH2, SUZ12 and EED throughout oocyte growth in wild-type mice using immunofluorescence (IF). EZH2 was detected in the oocyte nucleus of primordial to antral stage follicles, but SUZ12 and EED were detected only in primary and secondary follicle oocytes and not in primordial or antral follicle stage oocytes (Fig. 1a). Notably, co-expression of EED, EZH2 and SUZ12 in primary–secondary follicle oocytes occurred immediately before the expression of DNMT3L (DNA methyltransferase 3-Like), which marks the onset of de novo DNA methylation in growing oocytes (Fig. 1b), consistent with the initiation of H3K27me3 in oocytes prior to DNA methylation [44]. While EZH2 was detected in the nuclei of fully grown surrounded nucleolus (SN) GV oocytes, SUZ12 and EED were not (Fig. 1c). Although PRC2 has been detected in the cytoplasm of mature metaphase II (MII) oocytes [41–43], EED, EZH2 and SUZ12 were not detected on the chromosomes of MII, GV or Antral stage oocytes in this study even though they were readily detected in granulosa cells (Fig. 1c,d). However, all three PRC2 components were readily detected in maternal and paternal pronuclei of zygotes approximately 12 h (h) post-fertilisation (Fig. 1e). As embryonic activity of PRC2 does not occur until the 4-cell to morula stage [31, 34], the rapid recruitment of PRC2 to the pronuclei may reflect a supply of cytoplasmic PRC2 proteins [41–43] or could be derived from mRNAs in the mature oocyte. We cannot exclude the possibility that EED or SUZ12 protein below detection sensitivity were present on chromatin in antral, GV and MII oocytes. However, taken together, these data identify a transient window during which all three PRC2 components are present in primary–secondary oocytes and may therefore contribute to PRC2-dependent epigenetic programming immediately before genome-wide de novo DNA methylation and prior to the formation of GV oocytes.

### PRC2 is required for the repression of developmental genes in growing oocytes

**Fig. 2:**
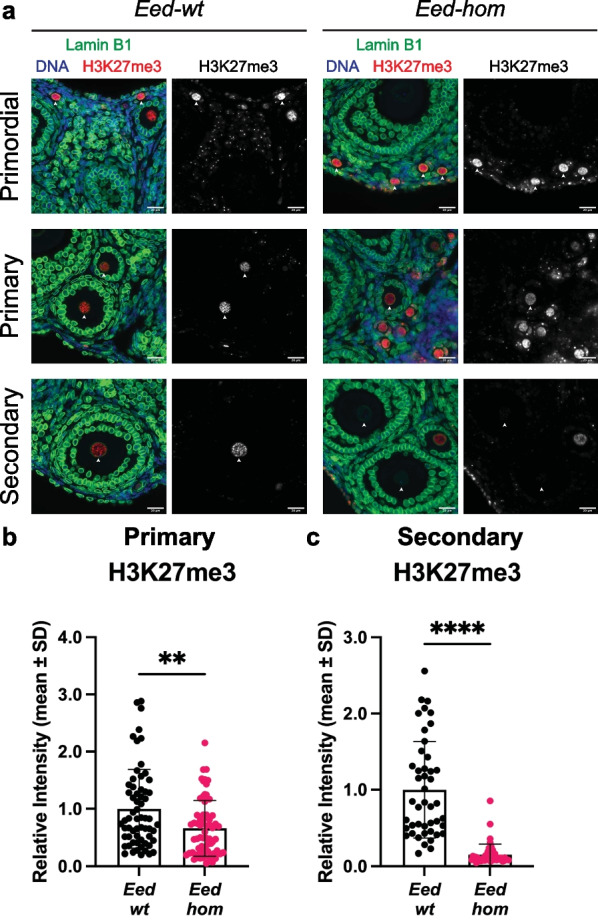
Deletion of *Eed* in oocytes reduced H3K27me3 in oocytes of primary and secondary follicles. **a** Representative images of H3K27me3 (red) immunostaining analysis in primordial (top), primary (middle) and secondary (bottom) follicle oocytes from *Eed-wt* and *Eed-hom* females. White arrowheads indicate the oocyte nucleus as defined by Lamin B1 (green). DAPI (blue) shows DNA in somatic cells. Images are representative of two ovaries from three biological replicates. Scale bars: 20 μm. **b–c** Quantification of H3K27me3 within oocyte nuclei of primary **(b)** and secondary **(c)** follicles from *Eed-wt* and *Eed-hom* females. Average intensity of *Eed-wt* was set to 1.0. **P < 0.005, two-tailed Mann–Whitney U test, *N* = 63 *Eed-wt* and 67 *Eed-hom* primary follicle oocytes. ****P < 0.0001, two-tailed Mann–Whitney U test, *N* = 45 *Eed-wt* and 47 *Eed-hom* secondary follicle oocytes. Error bars represent mean ± standard deviation

**Fig. 3:**
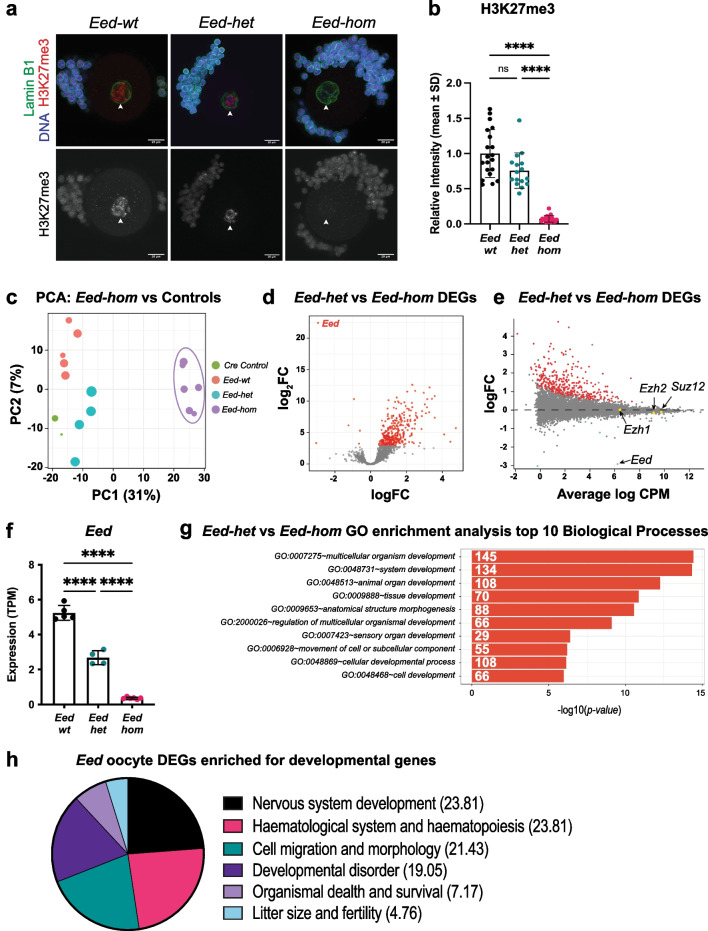
*Eed* is required for H3K27me3 establishment and developmental gene silencing in growing oocytes. **a,b** Representative images **(a)** and quantification **(b)** of H3K27me3 (red) IF in *Eed-wt*, *Eed-het*, and *Eed-hom* SN GV oocytes. White arrowheads indicate the oocyte nucleus as defined by Lamin B1 (green). DAPI (blue) shows DNA in somatic cells. Images represent 3–4 females per genotype, with 16–21 oocytes imaged per genotype. Scale bars: 20 μm. Average intensity of *Eed-wt* was set to 1.0. ****P < 0.0001, Kruskal–Wallis test plus Dunn’s multiple comparisons test, error bars represent mean ± standard deviation. **c** Principal Component Analysis (PCA) of RNA-seq data for *Eed-hom* (*n* = 6) vs *Eed-het* (*n* = 4), *Eed-wt* (*n* = 5) and *Eed-wt Cre* (*n* = 2) controls. **d–e** Differential gene expression analysis of *Eed-het* vs *Eed-hom* oocytes represented by volcano plot showing logFC against statistical significance and an MDplot showing logFC against average log counts per million reads. Genes with FDR-adjusted *P* < 0.05 are coloured in red. Deletion of *Eed* resulted in 349 significant DEGs (*Eed* oocyte DEGs), with 343 genes upregulated and 6 genes downregulated, including *Eed*. **f** *Eed* transcript levels (transcripts per million reads; TPM) in *Eed-wt*, *Eed-het* and *Eed-hom* GV oocytes. **g** GO enrichment analysis of *Eed* oocyte DEGs representing the top 10 significantly different biological processes impacted. **h** Pie chart displaying the proportion of significant pathways identified using Ingenuity pathway analysis

To investigate whether the transient activity of PRC2 in oocytes of primary–secondary follicles has functional importance, we deleted *Eed* using *Zp3Cre*, which leads to target gene excision specifically in oocytes from the primary follicle stage [40, 45]. Mating *Eed*^*fl/fl*^ females and *Eed*^*fl/*+^*;Zp3Cre* males yielded *Eed*^*fl/fl*^ (*Eed-wt*), *Eed*^*fl/*+^*;Zp3Cre* (*Eed-het*) and *Eed*^*fl/fl*^*;Zp3Cre* (*Eed-hom*) females. IF analysis demonstrated loss of EED staining in primary and secondary stage oocytes of *Eed-hom* samples, but not in *Eed-wt* samples (Additional file 1: Fig. S1). Moreover, EED staining was unaffected in granulosa cells, demonstrating that *Eed* deletion was oocyte-specific, as expected. Consistent with this, following the deletion of *Eed*, H3K27me3 was reduced by 35% in oocytes of primary follicles of *Eed-hom* relative to *Eed-wt* females (Fig. 2a-b). Depletion of H3K27me3 continued in secondary follicles (Fig. 2a, c), and was almost completely lost in fully grown GV oocytes, with 85% and 93% reductions in global H3K27me3 in *Eed-hom* oocytes compared to *Eed-wt* at these stages, respectively (Fig. 2a, c, 3a-b)*.* While H3K27me3 was significantly reduced in *Eed-hom* oocytes, it was not significantly reduced in *Eed-het* oocytes compared to *Eed-wt* controls (Fig. 3a–b).

To determine how the loss of H3K27me3 impacted oocyte transcription, we collected *Eed-wt*, *Eed-het, Eed-hom* and *Eed*^+*/*+^*;Zp3Cre* (*Eed*-*wt Cre* control) fully grown SN GV oocytes and performed RNA-seq. The proportion of SN GV oocytes in *Eed-hom* females was 65% compared to 58% in *Eed-wt* females (Additional file 1: Fig. S2), demonstrating that loss of EED and H3K27me3 did not detrimentally affect the formation of fully grown oocytes. Principal component analysis revealed that *Eed-hom* oocytes were transcriptionally distinct from *Eed-het* and *Eed*-*wt* oocytes (Fig. 3c). Comparison of gene expression in *Eed-wt* and *Eed-het* oocytes identified only two differentially expressed genes (*Mt1* and *Exoc)* as well as *Eed*, indicating that EED function in *Eed-wt* and *Eed-het* oocytes was similar. This was supported by our observation that H3K27me3 levels were not different in *Eed-wt* and *Eed-het* oocytes (Fig. 3b).

To determine if loss of *Eed* affected the expression of other PCR2 and PRC1 core components, we examined transcript levels of *Ezh1, Ezh2, Suz12, Ring1, Rnf2, Bmi1, Cbx1, Cbx2, Pcgf1 and Pcgf6*. Interestingly, *Ezh2, Suz12* and *Eed* were all transcribed at high levels in *Eed-wt* GV oocytes and *Ezh1* was transcribed at moderate levels (Additional file 1: Fig. S3a). As SUZ12 and EED proteins were undetectable in GV oocytes, but *Suz12* and *Eed* transcript levels were high , these data strongly indicate that *Eed* and *Suz12* are post-transcriptionally regulated in GV oocytes. This could occur post-transcriptionally through a mechanism such as microRNA mediated inhibition of *Eed* and *Suz12* mRNA translation or an undefined mechanism that post-translationally alters the structure of the EED and SUZ12 proteins, rendering them undetectable by the EED and SUZ12 antibodies used in this study. We propose that the former mechanism is more probable as it seems highly unlikely that post-translational masking of both the EED and SUZ12 proteins would occur via similar alterations as their protein sequences substantially differ. While *Eed* transcription was reduced to 51% in *Eed-het* oocytes and 6% in *Eed-hom* oocytes (Fig. 3f), there was no effect of *Eed* deletion on *Ezh1, Ezh2, Suz12,* or on transcription of any of the genes encoding PRC1 subunits in *Eed-het* or *Eed-hom* oocytes (Additional file 1: Fig. S3a-b). Moreover, while PRC2 expression in primary–secondary follicle oocytes preceded that of DNMT3L, transcription of *Dnmt3l, Dnmt3a* and *Dnmt3b* was not different in *Eed-hom* and *Eed-het* GV oocytes (Additional file 1: Fig. S3c), indicating that transcription of *Dnmt* genes was unaffected by PRC2 in GV stage oocytes.

In a past study, we mated wild-type males to females producing *Eed-wt, Eed-het* and *Eed-hom* oocytes created using *ZP3-Cre* deletion [40]. This demonstrated that homozygous deletion of *Eed* in oocytes caused a growth defect in *Eed* heterozygous experimental offspring, but heterozygous deletion of *Eed* in oocytes did not cause a similar growth defect in isogenic *Eed* heterozygous control offspring [40]. As these offspring were isogenic and sired by wild-type males, we concluded that the phenotype in offspring from *Eed-hom* oocytes was due to EED function in *Eed-hom* oocytes, but this was unaffected in *Eed-het* oocytes [40]. To identify genes that rely on EED for their epigenetic programming in oocytes and which may account for the genetically independent phenotype in offspring derived from *Eed-hom* oocytes, in this study we compared *Eed-hom* oocytes with *Eed-het* oocytes. This identified 349 genes that we termed *Eed* oocyte Differentially Expressed Genes (DEGs) as they were differentially expressed between *Eed-hom* and *Eed-het* oocytes (FDR < 0.05; Fig. 3d–e; Additional file 2: Table S1). Strikingly, 98% (343 genes) of the *Eed* oocyte DEGs were de-repressed, and only 2% (six genes), including *Eed,* were downregulated (Fig. 3d–f; Additional file 2: Table S1). As EED protein was only detected in primary–secondary follicle oocytes prior to the GV stage, these data strongly indicate that PRC2 establishes a repressive state in primary and secondary follicle oocytes that is maintained in GV oocytes.

Gene ontology (GO) and ingenuity pathway analyses (IPA) revealed that the *Eed* oocyte DEGs were strongly associated with foetal development, including the IPA categories of nervous system development (23.81%), haematopoiesis (23.81%), cell migration and morphology (21.43%) and developmental disorders (19.05%; Fig. 3g-h). Several genes involved in bone development, including *Prrx*, *Gli2*, *Sox5*, *Sox6*, *Hoxd9*, *Hoxd13*, *Bmp7*, *Sik3* and *Dcn* were also de-repressed (Additional file 2: Table S1). The neurogenesis and bone developmental genes are of interest as impaired skeletal and cognitive development are prominent features of Cohen-Gibson syndrome which results from de novo germline mutations in *EED* [21–23, 26]. Although 4.67% of the *Eed* oocyte DEGs were associated with “litter size and fertility”, categories associated with oocyte or ovarian development were not represented (Fig. 3g). Moreover, similar numbers of oocytes in *Eed-hom, Eed-het* and *Eed-wt* females indicated that oocyte growth and formation of fully grown oocytes was not impeded (Additional file 1: Fig. S2). Together, these observations strongly suggested that PRC2 establishes repressive H3K27me3 on a wide range of developmentally important genes during oocyte growth, > 95% of which were not primarily involved in oogenesis. Surprisingly, a comparison of the *Eed* oocyte DEGs with 209 transcription factors identified as direct target genes of PRC1 and PRC2 in embryonic stem cells [20] identified only 8 common genes (*Hoxd9, Hoxd13, Otx1, Lhx2, Six1, Nr2f2, Ovol1* and *Nfatc1*), indicating that the genes that were de-repressed on *Eed*-null oocytes were not typical polycomb target genes in ESCs (Additional file 2: Table S2).

### PRC2 regulates the establishment of H3K27me3 on developmental genes in growing oocytes

**Fig. 4:**
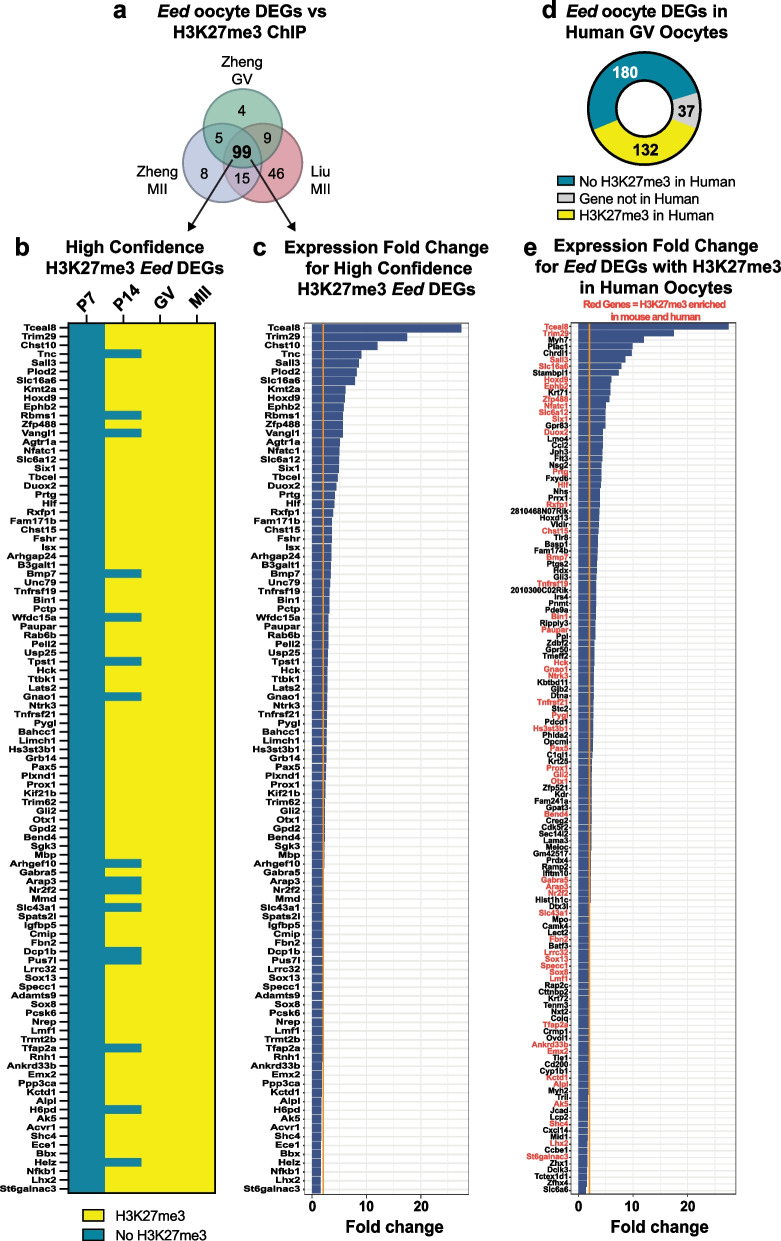
H3K27me3 is established on *Eed* oocyte DEGs in primary–secondary mouse oocytes and is conserved in human GV oocytes **a** Venn diagram showing *Eed* oocyte DEGs that contained H3K27me3 promoter peaks in GV and MII oocyte H3K27me3 ChIP-seq data sets [44, 46] identifying 99 “high-confidence” H3K27me3-enriched *Eed* oocyte DEGs. **b** Heat map showing promoter H3K27me3 enrichment status of 99 high-confidence H3K27me3-enriched *Eed* oocyte DEGs identified in P7, P14, GV and MII oocyte H3K27me3 ChIP-seq data sets from Liu et al., and Zheng et al*.,* [44, 46]. Blue: No H3K27me3 peaks, yellow: indicates presence of H3K27me3 peaks. **c** Expression fold change of the 99 high-confidence H3K27me3-enriched *Eed* oocyte DEGs (FDR < 0.05) in *Eed-hom* oocytes relative to *Eed-het*. The orange line indicates twofold change. **d** Donut chart showing the promoter H3K27me3 enrichment status of *Eed* oocyte DEGs in human GV oocytes [48]. Grey: Not conserved in humans, blue: no H3K27me3 peaks in promoter, yellow: H3K27me3 peak present in promoter. **e** Expression fold change of 132 mouse *Eed* oocyte DEGs (FDR < 0.05) that were H3K27me3-enriched in human GV oocytes. *Eed* oocyte DEGs commonly enriched for H3K27me3 in human and mouse GV oocytes are marked with gene names in red. The orange line indicates two-fold change. For **a**,**b** and** d**, promoter region was defined as 2000 bp upstream and downstream of TSS, overlap of > 200 bp H3K27me3 peaks with the promoter region was considered H3K27me3-enriched

To determine whether H3K27me3 was normally present in the promoters of *Eed* oocyte DEGs in GV oocytes we compared the *Eed* oocyte DEGs to H3K27me3 chromatin immunoprecipitation-sequencing (ChIP-seq) data sets from wild-type mouse GV and MII oocytes from Zheng and colleagues and Liu and colleagues [44, 46]. Of 349 *Eed* oocyte DEGs, the majority were identified both in the Zheng and Liu data sets (328 in Zheng, and 312 in Liu). Of the 328 genes from Zheng et al*.,* 111 (34%) and 127 (39%) had H3K27me3 peaks in GV and MII oocyte data sets, and 169 (52%) had H3K27me3 peaks in the Liu MII data set (Additional file 2: Table S3). Comparison of all data sets (our *Eed* oocyte DEGs with the Zheng GV and MII and Liu MII data sets) identified 99 DEGs with H3K27me3 in all three ChIP-seq data sets, which we defined as “high-confidence” H3K27me3-enriched oocyte DEGs (Fig. 4a; Additional file 2: Table S4). Of these 99 genes, comparison with data from a study of H3K27me3 in sperm [47] revealed that 88 also carried H3K27me3 on the paternal allele indicating that the majority of these genes are subject to H3K27 methylation in both the male and female germlines (Additional file 2: Table S4). While only ~ 30% of the *Eed* oocyte DEGs contained H3K27me3 under these highly stringent criteria, we propose that this is a conservative estimate given that low input ChIP-seq data were used, potentially limiting sensitivity compared to RNA-seq analysis. Supporting this, 114 H3K27me3-enriched DEGs overlapped between the Liu and Zheng MII data sets, indicating that not all genes with H3K27me3 were consistently detected in the ChIP-seq analyses.

Gradual enrichment of H3K27me3 has been demonstrated in growing oocytes [44]. Using these additional ChIP-seq data sets [44], we next investigated at what stage H3K27me3 peaks were established at the high-confidence H3K27me3-enriched oocyte DEGs by determining the H3K27me3 state at the promoter regions of these genes in post-natal day (P)7 (primary) and P14 (secondary) growing oocytes. None of the 99 high-confidence H3K27me3-enriched oocyte DEGs had H3K27me3 peaks in primary (P7) oocytes, but 83 had H3K27me3 peaks in secondary (P14) oocytes and all 99 DEGs had H3K27me3 in GV and MII oocytes (Fig. 4b; Additional file 2: Table S4). Accordingly, each of these genes was expressed at significantly higher levels in *Eed-hom* compared to *Eed-het* GV oocytes (FDR < 0.05; Fig. 4c). Collectively, these data demonstrate that H3K27me3 was established within the promoters of developmental genes during the window in which all three core components of PRC2 were detected in primary–secondary follicle oocytes and that EED was required for their repression given that these genes were de-repressed in *Eed*-null GV oocytes.

### PRC2 establishment of H3K27me3 at developmental genes is conserved in human oocytes

To understand whether the mouse *Eed* oocyte DEGs were also enriched for H3K27me3 in human oocytes, we examined published H3K27me3 data from human GV oocytes [48], defining promoter regions 2000 bp upstream–2000 bp downstream of the TSS as we did for the mouse data sets. Of 349 *Eed* oocyte DEGs, 37 were excluded as “not conserved in human”, including 25 predicted genes, RIKEN transcripts or pseudogenes (Fig. 4d; Additional file 2: Table S3). Of the 312 remaining *Eed* oocyte DEGs, 132 contained H3K27me3 in their promoters in human GV oocytes (Fig. 4d; Additional file 2: Tables S3, S5). Of the 132 *Eed* oocyte DEGs containing H3K27me3 in human GV oocytes, 79 and 54 also contained H3K27me3 in the mouse MII and GV data sets generated by Liu et al*.* and Zheng et al*.*, respectively (Additional file 2: Table S5 [44, 46]). Moreover, all 132 *Eed* oocyte DEGs identified as H3K27me3-enriched in human were expressed at significantly higher levels in *Eed-hom* compared to *Eed-het* GV oocytes (FDR < 0.05; Fig. 4e). In common with the lack of nuclear-localised EED or SUZ12 protein in mouse GV oocytes (Fig. 1c), human GV oocytes lack *EED* and *SUZ12* transcripts [48]. Together, with the observation that *Eed* oocyte DEGS are almost exclusively de-repressed with the loss of EED and H3K27me3, these data strongly indicate that H3K27me3 establishment occurs on *Eed* oocyte DEGs prior to the GV stage in both human and mouse growing oocytes.

### *Eed* oocyte DEGs include non-imprinted autosomal, imprinted and X-linked genes

**Fig. 5:**
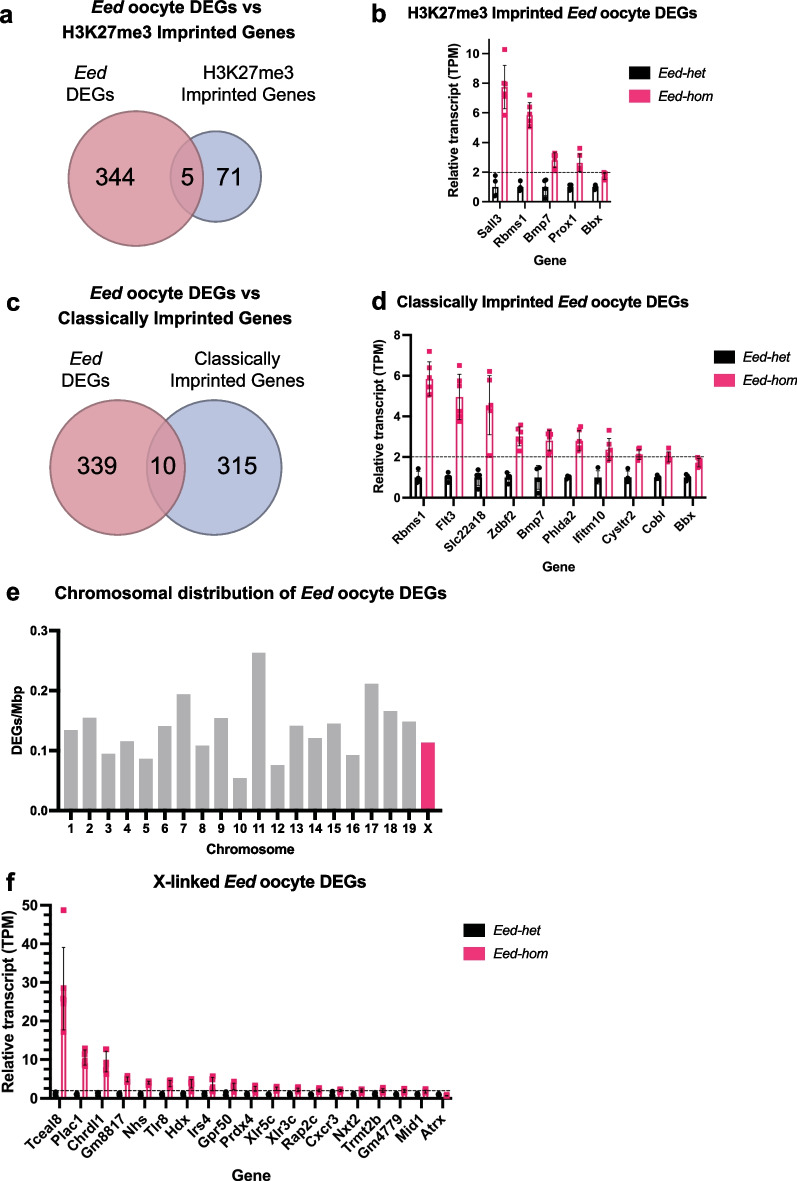
EED is required for repressing a wide range of genes in growing oocytes that are not canonically or non-canonically imprinted or X-linked genes **a** Venn diagram comparing *Eed* oocyte DEGs against putative H3K27me3 imprinted genes [35]. **b** Expression of putative H3K27me3-imprinted *Eed* oocyte DEGs in *Eed-hom* oocyte relative to *Eed-het*. Data represent the mean transcripts per million (TPM), with *Eed-het* mean set to 1.0. **c** Venn diagram comparing *Eed* oocyte DEGs against known or predicted classically imprinted genes [49, 50]. **d** Expression of known or predicted classically imprinted *Eed* oocyte DEGs in *Eed-hom* oocyte relative to *Eed-het*. Data represent the mean transcripts per million (TPM), with *Eed-het* mean set to 1.0. **e** graphical representation of *Eed* oocyte DEGs per mega base vs chromosome for autosome and the X-chromosome. **f** Expression of X-linked *Eed* oocyte DEGs in *Eed-hom* oocyte relative to *Eed-het*. Data represent the mean transcripts per million (TPM), with *Eed-het* mean set to 1.0

As EED regulates DNA methylation-independent H3K27me3 imprinting in oocytes, we compared the *Eed* oocyte DEGs against a previously published list of 76 putative H3K27me3-imprinted genes [35]. Of the 349 *Eed* oocyte DEGs we identified in GV oocytes, five (*Bbx, Bmp7, Rbms1, Sall3* and *Prox1*) were putative H3K27me3 imprinted genes (Fig. 5a; Additional file 2: Table S6). These genes were all expressed at significantly higher levels in *Eed-hom* compared to *Eed-het* GV oocytes (FDR < 0.05; Fig. 5b; Additional file 1: Fig. S4a), demonstrating that EED is required for their repression in oocytes. Of interest, analysis of a sperm ChIP-seq data set ​​[47] revealed that all five genes also contained H3K27me3 on the paternal allele, indicating that these genes have similar H3K27me3 signatures in male and female gametes (Additional file 2: Table S3). Of these five genes, paternally biased expression was observed for three in androgenetic morula (*Bbx, Bmp7* and *Rbms1*) and one in blastocysts (*Rbms1*) [35]. Notably, Inoue et al. identified five H3K27me3-imprinted genes (*Gab1, Phf17, Sfmbt2, Slc38a4 and Smoc1*) that maintain paternal-biased expression in the epiblast, visceral endoderm, extraembryonic ectoderm and/or E9.5 placenta [35]. Loss of imprinting at either *Slc38a4* or micro-RNAs within *Sfmbt2* has been functionally associated with placental hyperplasia in SCNT-derived offspring [36, 38]. In addition, *Smoc1* and *Gab1* have also been implicated in this phenotype [37]. However, none of these genes were *Eed* oocyte DEGs (Additional file 2: Table S6).

In order to determine whether any *Eed* oocyte DEGs were imprinted genes, we also compared the 349 *Eed* oocyte DEGs to 325 known or predicted classically imprinted genes in mice [49, 50]. Ten *Eed* oocyte DEGs were identified as imprinted genes (FDR < 0.05; Fig. 5c; Additional file 2: Table S6; *Zdbf2, Ifitm10, Phlda2, Rbms1, Bmp7, Bbx, Flt3, Slc22a18, Cobl* and *Cysltr2*). As with the putative H3K27me3-imprinted genes, these were all upregulated in *Eed-hom* oocytes (Fig. 5d; Additional file 1: Fig. S4b) indicating that EED is required to silence these genes in oocytes. Analysis of the sperm data set ​​[47] revealed that all of these genes contained H3K27me3 on the paternal allele in sperm and three of these genes (*Bbx, Bmp7* and *Rbms1*) were also included on the H3K27me3-imprinted gene list (Additional file 2: Table S3). In human GV oocytes *Bmp7, Sall3, Prox1, Zdbf2, Phlda2, Flt3* and *Ifitm10* also carried H3K27me3, but *Bbx, Rbms1, Cysltr2* and *Slc22a4* did not.

In mice, X-inactivation is initiated after fertilisation from the 2–4 cell stage and is restricted to the paternal X-chromosome in pre-implantation offspring prior to the establishment of random X-inactivation in embryonic cells after implantation. While PRC2 regulates X-inactivation in pre-implantation embryos and somatic cells of post-implantation embryos, the inactive X is reactivated in XX primordial germ cells and both X-chromosomes are active in growing oocytes [51–53]. To determine whether there was any bias in gene silencing across the autosomes and the X-chromosome in EED-deficient oocytes we determined the relative representation of the *Eed* oocyte DEGs across all chromosomes. However, the representation of the oocyte DEGs across the autosomes and X-chromosome was similar, with 19 of 349 genes X-located and no substantial bias towards the X-chromosome or particular autosomes (Fig. 5e; Additional file 2: Table S7). As with most genes on the autosomes, of the 19 X-linked genes identified, 18 were expressed at higher levels in *Eed-hom* compared to *Eed-het* GV oocytes (FDR < 0.05; Fig. 5f; Additional file 1: Fig. S4c) demonstrating that EED and H3K27me3 contribute to silencing individual X-linked genes in the absence of X inactivation in oocytes.

Together, these data indicated that of the 349 *Eed* oocyte DEGs identified, 12 were imprinted genes, 19 were located on the X-chromosome and the remaining 328 were non-imprinted autosomal genes, many of which are known to regulate development.

### LINE-1 transposable elements were not de-repressed in *Eed*-null oocytes

**Fig. 6:**
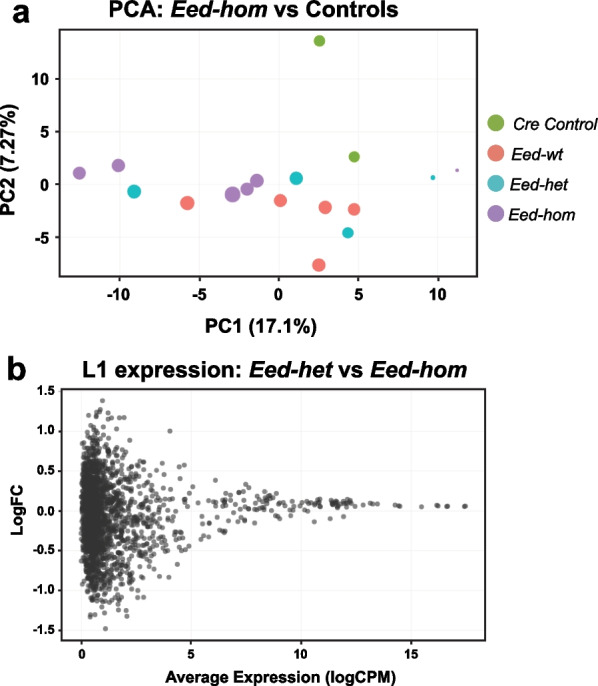
Loss of *Eed* in growing oocytes did not impact expression of LINE-1 transposons. **a** Principal Component Analysis (PCA) of RNA-seq data for L1 elements in *Eed-hom* (*n* = 6) vs *Eed-het* (*n* = 4), *Eed-wt* (*n* = 5) and *Eed-wt Cre* (*n* = 2) controls. **b** Differential expression analysis of L1 elements for *Eed-het* versus *Eed-hom* females

Previous studies have proposed and/or demonstrated a link between H3K27me3 and the repression of transposable elements (TEs) in foetal germ cells and embryonic stem cells when DNA methylation levels are low during epigenetic reprogramming [54–57]. To determine whether the transcription of transposable elements was affected in oocytes lacking EED, we demasked repeat sequences in our RNA-seq data and analysed the expression of LINE-1 (L1) elements. The total input reads indicated that similar percentages of L1 element reads were aligned in *Eed-hom*, *Eed-het* and *Eed-wt* oocytes (Additional file 1: Fig. S5a). Moreover, regardless of whether elements were mapped uniquely or multi-mapped, read totals indicated similar expression levels for L1s in oocytes of all genotypes (Additional file 1: Fig. S5b). Analysis of the extent to which multiple reads occurred revealed that the majority of reads mapped 1, 2 or 3 times (accounting for ~ 80% of all reads), while around 10% mapped 4–5 times and the remaining reads mapped 5–20 times, with no differences in mapping between genotypes (Additional file 1: Fig. S5c). Principal component analysis revealed overlapping clusters for samples for all genotypes and no differentially expressed L1 elements were identified based on a threshold of FDR < 0.05 (Fig. 6A, B). Together these data indicate that loss of H3K27me3 in GV oocytes did not substantially alter L1 expression.

### *Eed* oocyte DEGs were not dysregulated in pre-implantation embryos

Our observations revealed a highly specific window during which all three components of PRC2 were present in primary–secondary oocytes and identified a role for EED in establishing H3K27me3 on a wide range of developmentally important genes in primary–secondary follicle oocytes. As > 95% of these genes are not known to regulate oocyte development, yet EED is required for their repression in oocytes, we proposed that loss of EED-dependent repression of these genes in oocytes may result in their de-repression in pre-implantation embryos. To determine if this was the case we re-analysed RNA-seq data from morula and blastocysts derived from *Gdf9Cre Eed*-null [33] and *Zp3Cre Eed*-null oocytes [34]. The *Eed-*deleted oocyte-derived morulae contained 128 DEGs (P < 0.05, all with FDR ~ 1.0; Additional file 2: Table S8) and the blastocysts contained 400 DEGs (P < 0.05; FDR < 0.05; Additional file 2: Table S9) compared to their *Eed*-*wt* oocyte-derived counterparts. Although all genes identified in morulae from *Eed-wt* and *Eed-hom* oocytes had an FDR > 0.05, of the 128 genes identified with a p < 0.05, six were *Eed* oocyte DEGs including *Plxnd1, Tceal8, Rap2c, Bbx, Xlr3c* and *Trmt2b* (Additional file 1: Fig. S6). In addition, five *Eed* oocyte DEGs were dysregulated in blastocysts (*Chrdl1, Lonrf2, Trim6, Cyp1b1* and *Ccbe1*; Additional file 1: Fig. S3). With the exception of *Tceal8* and *Bbx*, all were downregulated in pre-implantation embryos derived from *Eed-*null oocytes (Additional file 2: Tables S8-S9). Only two genes were commonly downregulated in the morulae and blastocyst data sets (*Tspan6* and *Gk*) and no genes were dysregulated in all three data sets (Additional file 1: Fig. S3). Similarly, there were no genes in the morula DEGs and only seven genes (*Zfpm2, Pax9, Tbx4, Foxc2, Atoh8, Msx1* and *Vsx1*) in the blastocyst DEGs that were common with 209 genes identified as direct Polycomb target transcription factors in ESCs (Additional file 2: Table S10; [20]).

To determine whether H3K27me3 was maintained at *Eed* oocyte DEGs in pre-implantation embryos, we used CUT&RUN data revealing the H3K27me3 state on the maternal and paternal alleles in morula stage embryos (Additional file 2: Table S3) [33]. Six *Eed* oocyte DEGs contained H3K27me3 on the maternal, but not the paternal allele in morula. Of the remaining 343 *Eed* oocyte DEGs, 322 were devoid of H3K27me3 on both the maternal and paternal alleles. The maternal and paternal alleles were not distinguishable for 21 genes in the source data set [33]. These data indicate that H3K27me3 is normally cleared from the vast majority of *Eed* oocyte DEGs in morula stage embryos.

## Discussion

In this study, we identified a discrete period during which all three core components of PRC2 co-localised to the nucleus in primary–secondary follicle oocytes, that H3K27me3 was established on a wide range of developmental genes in this window and that EED was required to repress these genes in GV oocytes. This transient activity of PRC2 facilitated H3K27me3 establishment on a wide range of *Eed* oocyte DEGs immediately prior to DNMT3L upregulation, indicating that EED-dependent programming precedes de novo DNA methylation and that epigenetic programming is highly temporally and spatially regulated during oocyte growth. As many of the same genes were marked by EED-dependent H3K27me3 in mouse and human GV oocytes, the role of PRC2 appears to be conserved in human and mouse oocytes. These findings broaden the understanding of the temporal, spatial and functional activity of PRC2 in the female germline.

The *Eed* oocyte DEGs identified included five H3K27me3 imprinted and seven classically imprinted genes (three of the classically imprinted genes detected were also listed as H3K27me3 imprinted genes). However, the vast majority of *Eed* oocyte DEGs were not known imprinted loci, but included many genes known to regulate cell differentiation during foetal development. Despite this, the *Eed* oocyte DEGs identified were not over-expressed in pre-implantation offspring and lost H3K27me3 during pre-implantation development. While the significance of PRC2 regulation of these genes requires further investigation, previous studies revealed that loss of EED in the oocyte affects early development and post-natal outcomes in opposing ways: offspring from *Eed-*null oocytes exhibit both placental and offspring growth restriction at E10.5 [33], but were subsequently overgrown by early post-natal stages [40]. While the mechanisms remain unclear, the collective data indicate that PRC2 acts at multiple levels of oocyte growth and pre-implantation development to modulate outcomes in offspring.

In previous studies, there has been a significant focus on PRC2 as a maternal factor complex that regulates aspects of pre-implantation development, including X-inactivation [31–34, 58]. In this study, we detected EZH2 in oocytes from the primordial follicle stage through to the SN GV oocyte stage, whereas SUZ12 and EED were only identified within primary and secondary follicle stage oocytes. Since all three components are required for PRC2 methyltransferase activity [9–12], this identifies a discrete window in primary–secondary follicle oocytes during which PRC2 has the capacity to catalyse methylation of H3K27. While proteins for EED or SUZ12 were not detected in association with chromatin of GV and MII oocytes in our study, all three proteins were robustly detected in both maternal and paternal pronuclei 12 h post-fertilisation, highlighting a role for maternally inherited PRC2 in zygotes. As SUZ12, EED and EZH2 proteins were detected in MII oocytes using western blots in previous studies [41, 42], it is likely that a maternal supply of protein resides in the cytoplasm but is difficult to detect using IF. However, as *Eed* and *Suz12* were also transcribed at high levels in GV oocytes, post-transcriptionally regulated maternal RNA for *Eed, Suz12* and *Ezh2* may be inherited via the oocyte and translated after fertilisation to facilitate the enrichment of PRC2 in the maternal and paternal pronuclei of zygotes. Regardless of the mode of inheritance, these observations suggest that PRC2 has distinct activities that differentially impact epigenetic regulation in growing oocytes and pre-implantation development in offspring.

Consistent with this, loss of EED in the oocyte affected different gene sets in oocytes and in pre-implantation offspring. While < 5% of *Eed* oocyte DEGs were imprinted genes, the vast majority were not imprinted and many were associated with post-implantation cell differentiation and tissue development. Moreover, although EED was required for the repression of these genes in oocytes, very few *Eed* oocyte DEGs were dysregulated in morula and blastocysts derived from *Eed-*null oocytes, and H3K27me3 was lost on these genes during pre-implantation development. While this is consistent with previous findings that many developmental genes lose H3K27me3 during pre-implantation development [44], it also indicates that their repression at this stage does not require H3K27me3. Despite this, PRC2 is required in the oocyte for regulating a large cohort of genes in pre-implantation embryos that are distinct from the *Eed* DEGs identified here, and are dysregulated when *Eed* is deleted in oocytes. Thus, while EED is required for enriching H3K27me3 and repressing a large cohort of developmental genes in primary–secondary oocytes, it also regulates a distinct set of genes in pre-implantation embryos. Therefore, PRC2 functions both to epigenetically programme a wide range of imprinted and non-imprinted genes in oocytes and acts as a maternal factor in the zygote.

**Fig. 7:**
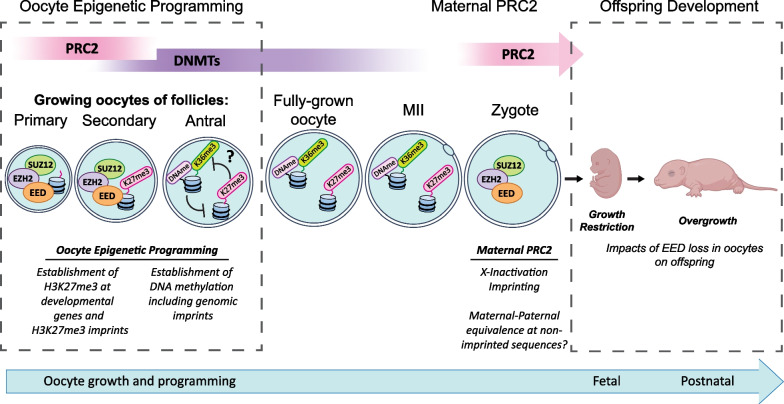
Summary of PRC2 functions during oocyte growth and maturation and pre-implantation development. All three essential components of PRC2 are present in growing oocytes only at the primary to secondary stages and establish H3K27me3 on H3K27me3-imprinted genes and a wide range of developmental genes, potentially programming *Eed* oocyte DEG expression in offspring. As this activity immediately precedes de novo DNA methylation, we propose that H3K27me3 established prior to DNA methylation may act as a “place-keeper” protecting developmental genes from modifications such as H3K36me3 and/or DNA methylation. Cytoplasmic PRC2 proteins and/or mRNA are inherited via the mature oocyte and regulate pre-implantation development, including X-inactivation, H3K27me3-dependent imprinting [31, 34, 35] and establishment of maternal–paternal equivalence at non-imprinted sequences. Loss of PRC2 in the oocyte leads to embryo growth restriction [33] but offspring are ultimately overgrown immediately after birth [40]

Our data indicates that H3K27me3 was established in the promoters of *Eed* oocyte DEGs immediately before the onset of DNA methylation, raising the possibility that H3K27me3 could influence the establishment of other epigenetic modifications in oocytes. While H3K27me3 and DNA methylation are generally mutually exclusive [44], H3K36me3 coincides with DNA methylation in oocytes [59] and H3K36me3 deposition increases in regions that subsequently acquire DNA methylation in oocytes [60]. Further, while loss of H3K36me3 in oocytes results in ectopic H3K27me3 deposition in oocytes [59], it is not known whether H3K36me3 is altered in oocytes following the loss of H3K27me3. As the relationship between DNA methylation/H3K36me3 and H3K27me3 is antagonistic, a potential role of H3K27me3 in oocytes may be to act as a “place-keeper” that ensures the promoters of EED-dependent oocyte genes are not subject to other forms of epigenetic alteration (Fig. 7), such as DNA methylation or H3K36 methylation [59]. Such an effect may be similar to that proposed for H3K27me3 in protecting regions from DNA methylation during sperm maturation [47] and may explain both the enrichment of H3K27me3 on genes that regulate foetal development and why H3K27me3 is cleared from these genes during pre-implantation offspring development.

As transient PRC2 activity occurs early during oocyte growth and immediately precedes DNMT activity [61], it is reasonable to speculate that in the absence of H3K27me3 *Eed* oocyte DEGS may accumulate H3K36 and subsequent DNA methylation. Aberrant establishment of DNA methylation in *Eed-*null oocytes may then contribute to developmental outcomes in subsequent offspring. To our knowledge, no study has directly measured the impact of maternal PRC2 deletion on global DNA methylation within the oocyte. Inoue et al*.* observed that classically imprinted gene expression was normal in maternal *Eed-*null embryos and concluded that DNA methylation establishment at classically imprinted genes was not impacted by *Eed* deletion [33]. However, this does not exclude the possibility that H3K27me3 protects other regions from establishing DNA methylation in oocytes, particularly the oocyte DEGs identified in this study and other H3K27me3-enriched genes identified in other studies [33, 35, 44, 46]. Further work is therefore required to determine the potential impact of H3K27me3 loss in primary–secondary oocytes on the broader epigenetic landscape of mature oocytes.

The potential for H3K27me3 to guide the epigenetic state of target genes in oocytes is of interest, as using this model we previously observed post-natal overgrowth in offspring derived from oocytes lacking EED [40]. However, another study showed that E10.5 embryos from *Eed*-null oocytes were growth-restricted [33], indicating that loss of EED in oocytes differentially impacts embryonic and offspring growth. Placental hyperplasia has been attributed to the loss of H3K27me3-dependent imprinting at a small number of genes in mice derived by SCNT [36–38], but is considered to be a placental effect. While this could lead to large offspring from oocytes lacking EED, it is yet to be observed in a model with oocyte-specific *Eed* deletion and does not explain why early embryos were smaller. One explanation could be that loss of maternal PRC2 in the zygote and early pre-implantation embryo hampers early development. However, an alternative explanation is that EED and H3K27me3-dependent programming of developmental genes in growing oocytes may also affect offspring growth and development through an as-yet-undefined epigenetically inherited mechanism, such as altered DNA methylation.

Finally, previous studies have demonstrated a link between H3K27me3 and repression of repetitive sequences when DNA methylation levels are low, including in male and female foetal germ cells and embryonic stem cells [55–57]. However, we did not observe any change in L1 expression in GV oocytes. With the obvious caveat that these sequences may have been repressed by other epigenetic modifications such as DNA methylation in growing oocytes, our data indicate that PRC2 is dispensable for repressing L1 sequences, and possibly other repetitive sequences in GV oocytes.

## Conclusions

In summary, we provide evidence that PRC2 acts transiently to establish H3K27me3 on a wide range of developmental genes in primary–secondary follicle oocytes and that this activity is required for the repression of these genes in fully grown oocytes. As this activity precedes DNA methylation, and loss of H3K36me3 allows inappropriate spreading of H3K27me3 in oocytes, it seems likely that loss of H3K27me3 will affect other epigenetic programming events in oocytes. Moreover, as the transient activity of PRC2 in primary to secondary oocytes is distinct from the established maternal factor activity of PRC2 in pre-implantation embryos, and different gene sets are affected in oocytes and in pre-implantation embryos, these activities of PRC2 have distinct developmental consequences in offspring. Finally, as common genes are targeted for H3K27me3 enrichment in both mouse and human oocytes, understanding the activity of PRC2 during the growth of murine oocytes is likely to provide insight not only into non-genetic inheritance, but also for determining how altered PRC2 activity in oocytes affects human health.

## Methods

### Mouse strains, animal care and ethics

Mice were housed at Monash Medical Centre Animal Facility using a 12-h light–dark cycle as previously reported [40]. Food and water were available ad libitum with room temperature maintained at 21–23 °C with controlled humidity. All animal work was undertaken in accordance with Monash University Animal Ethics Committee (AEC) approvals. Mice were obtained from the following sources: *Zp3Cre* mice (C57BL/6-Tg 93knw/J; Jackson Labs line 003,651, constructed and shared by Prof Barbara Knowles [62]), *Eed* floxed mice (*Eed*^fl/fl^) (B6; 129S1-*Eed*tm1Sho/J; Jackson Labs line 0,022,727; constructed and shared by Prof Stuart Orkin [63]. The *Eed* line was backcrossed to a pure C57BL6/J and shared with us by Associate Professor Rhys Allen and Professor Marnie Blewitt, Walter and Eliza Hall Institute for Medical Research, Melbourne.

### Genotyping

All animals were genotyped via ear punch at weaning by Transnetyx (Cordova, TN) using real-time PCR assays (details available upon request) designed for each gene as described previously [40].

### Collection, antibody incubation and detection of ovaries for immunofluorescence

Ovaries for immunofluorescence (IF) were fixed in 4% paraformaldehyde (PFA) overnight at 4 °C. Samples were then washed in PBS and processed through 70% ethanol and embedded in paraffin blocks, sectioned at 5 μm and transferred to Superfrost™ Plus slides (Thermo Fisher). Antigen retrieval was performed using DAKO citrate buffer (pH 6.0) at 98 °C for 30 min, and non-specific binding was blocked in PBS containing 5% BSA and 10% donkey serum for 1 h at RT. The blocking solution was replaced with PBS containing 0.1% Triton X-100 (PBSTX) and appropriately diluted primary antibodies (Supp. Materials and Methods) and incubated overnight (o/n) at 4 °C. Slides were washed in PBS and incubated with PBSTX containing secondary antibody for 1 h at RT. After the final washes in PBS, slides were rinsed in distilled H2O, mounted in DAPI ProLong Gold® and dried overnight. Fluorescence was detected using the VS120 Slide Scanner and quantified using QuPath Image Analysis Software (QuPath). Background fluorescence in the oocyte cytoplasm was removed from the nuclear intensity. When comparing control versus experimental groups, the control mean was set to 1.0.

### Collection of oocytes and pre-implantation embryos for immunofluorescence

Ovaries were punctured using 30 G needles to release oocytes. GV oocytes were partially denuded mechanically using a narrow-bore glass pipette. For MII oocyte and zygote collections, females were injected with 5 international units (IU) of pregnant mare serum gonadotropin (PMSG) followed by human chorionic gonadotropin (hCG; 48-h interval) and in the case of zygote collections were bred to C57BL/6 males. MII oocytes or zygotes were removed from the ampulla and denuded in M2 media containing hyaluronidase (0.3 mg/ml). Samples were fixed in 4% PFA containing 2% Triton X-100 for 30 min at RT. Samples were then washed in PBS containing 0.1% Tween, 0.01% Triton X-100 and 1% BSA (PBST-BSA) and stored in PBST-BSA.

### Oocyte and zygote whole-mount immunofluorescence

GV, MII oocytes and zygotes were blocked in PBST-BSA containing 10% donkey serum for 1 h at RT. The solution was then replaced with PBST-BSA containing appropriately diluted primary antibodies (Supp. Materials and Methods) and incubated o/n at 4 °C. Samples were washed in PBST-BSA and then incubated in PBST-BSA containing secondary antibodies (Supp. Materials and Methods) for 4 h (GVs) or 1 h (MII and zygote) in the dark at RT. After washing with PBST-BSA, samples were incubated with Hoechst 33342 (500 µg/ml) or DAPI (100 µg/ml) for 1 h at RT, washed and stored in PBST-BSA until imaging. Fluorescence was detected using the Nikon C1 inverted confocal microscope, and signal intensity was quantified using ImageJ. Background fluorescence levels were measured in the cytoplasm and removed from nuclear intensity, with the control mean set to 1.0 for comparisons.

### Collection of oocyte RNA and RNA-sequencing

Cumulus-oocyte complexes (COCs) were collected from 8- to 12-week-old female mice and transferred to M2 media. Oocytes were denuded mechanically with a narrow-bore glass pipette and incubated with M2 media containing 5 μg/mL Hoechst 33342 for 10 min at 37 °C. GV oocytes were then scored as either surrounded nucleolus (SN), or non-surrounded nucleolus (NSN) based on Hoechst staining. SN oocytes were then collected, frozen on dry ice and stored at -80 °C until RNA extraction. Ten to fifteen oocytes isolated from each female were pooled, and total RNA was isolated using the Agencourt RNAdvance Cell V2 extraction kit. High-quality RNA (RIN > 7.5) was used for library preparation (> 1.2 ng total RNA) using the Nugen Trio Library protocol, MU01440V2; 2017. 75 bp single-end sequencing was carried out on 4–6 libraries/genotype using Illumina NextSeq500 High output mode and v2.5 chemistry (Illumina Protocol 15,046,563 v02, Mar 2016) to collect > 25 M reads per sample.

### RNA-sequencing data analyses

Adaptor and low-quality sequences in raw sequencing reads were trimmed using AdaptorRemovel [64] (v2.2.1) with the following parameters: –trimns –trimqualities –minquality 20 –minlength 35. Clean reads were mapped to the mouse reference genome (GRCm38) using STAR [65] (v2.5.3a) with default settings. Raw counts for mouse reference genes (ensembl-release-93) were calculated using featureCounts [66] (v1.5.2) based on mapped bam files with the following parameters: -Q 10 -s 2. “Voom” method was used to estimate the mean–variance relationship and generate precision weights for samples, and then differential gene expression analysis was carried out using R package. Differential gene expression analysis was carried out using R package “limma” [67] with “treat” function with parameter as “lfc = log(1.2)”. Statistically significantly differentially expressed genes were identified using “FDR < 0.05”. Gene ontology (GO) enrichment analysis for significantly differentially expressed genes was carried out using The Database for Annotation, Visualisation and Integrated Discovery (DAVID) with the following settings: GO term level 3, minimum gene count 5, and FDR < 0.05 [68].

### Analyses of genome-wide H3K27me3 distribution and H3K27me3 data sets in oocytes

*Eed* GV DEGs were compared to publicly available H3K27me3 ChIP-seq, CUT & RUN and CUT & TAG data sets of oocytes, sperm and pre-implantation embryos. The data sets used are summarised in Supp. Materials and Methods.

For the data set from Zheng et al*.* [44] (GSE76687), processed files including whole genome scale broad H3K27me3 peaks were downloaded and used for the comparison. For the data set from Liu et al*.* [46] (GSE73952), H3K27me3 states of the promoter regions of mouse reference genes were retrieved from Additional file 2: Table S1 of the paper and used for the comparison. For the data set from Erkek et al*.* [47] (GSE42629), raw sequencing data were downloaded and then adaptor and low-quality sequences were trimmed using bbduk (v38.94). Clean reads were mapped to the mouse reference genome (mm9) using bowtie2 [69] (v2.4.4) with default settings. H3K27me3 peaks were identified using MACS2 [70] (v2.1.1) with the following parameters: -g 1.87e9 –nomodel –broad -q 0.05. For the data set from Inoue et al*.* [33] (GSE116713), bigwig format files were downloaded and converted to bedGraph format using “bigWigToBedGraph” from UCSC utilities, and then H3K27me3 peaks were called using MACS2 (v2.1.1) based on the bedGraph files with the following parameter: -c 1.3 (equivalent to P value < 0.05). For the human data set from Xia et al*.* 2019 (GSE124718), processed files including whole genome scale broad H3K27me3 peaks were downloaded and used for the comparison [48].

This comparison took the 349 *Eed* GV DEGs and asked whether their promoters (defined as 2 Kb up- or downstream of TSS to be consistent with Liu et al*.* 2016) overlapped with H3K27me3 peaks (> 200 bp overlap) in the above-mentioned publicly available H3K27me3 ChIP or CUT & RUN data sets. For the human data set from Xia et al. 2019 (GSE124718), human orthologous genes of mouse 349 *Eed* GV DEGs were identified and used for the comparison.

### Statistical analyses

GraphPad Prism 9 was used for statistical analysis and to graph data sets. As appropriate, parametric Student’s *t* tests or ANOVA or nonparametric equivalents as indicated in figure legends.

## Supplementary Information

**Additional file 1: Fig. S1:** Zp3Cre-mediated Eed deletion results in loss of EED in oocytes from the primary follicle stage in Eed-hom females. Representative images of EED (red) immunofluorescence analysis in primary (top), secondary (middle) and antral (bottom) follicles in females producing *Eed*-wt and *Eed*-hom oocytes. a. shows widefield follicle images and b. shows magnified images of the area inside the white squares containing the oocyte nucleus. Lamin B1 (green) marks the nuclear lamina and delineates the edge of the oocyte nucleus in b. DAPI (blue) shows DNA in somatic cells. Images are representative of two ovaries from three biological replicates. Scale bars: 20 μm. **Fig. S2:** Deletion of *Eed *in oocytes moderately increased the rate of Surrounded Nucleolus (SN) GV oocytes compared to Non-Surrounded Nucleolus (NSN) GV oocytes. Percentage of SN (left) and NSN (right) GV oocytes obtained from *Eed*-wt, *Eed*-het, and *Eed*-hom females during oocyte collections. *P < 0.05, one-way ANOVA plus Tukey’s multiple comparisons test, N = 7 *Eed*-wt, 5 *Eed*-het and 7 *Eed*-hom females. Error bars represent mean ± standard deviation. **Fig. S3:** Loss of *Eed *in growing oocytes did not impact the transcription of genes encoding other core PRC2 subunits, PRC1 core components or DNMTs. Expression of core (a) PRC2 and (b) PRC1 components, and (c) DNMTs in *Eed*-wt, *Eed*-het and *Eed*-hom oocytes. Data represent the mean transcripts per million reads (TPM) for each gene from N = 5 *Eed*-wt, 4 *Eed*-het and 6 *Eed*-hom females. FDR < 0.05 for *Eed* only, error bars represent mean ± standard deviation. **Fig. S4:** *Eed* deletion results in up-regulation of a subset of imprinted and X-linked genes in *Eed*-hom GV oocytes. Expression of core (a) putative H3K27me3-imprinted (b) classically imprinted, and (c) X-linked *Eed* oocyte DEGs in *Eed*-het and *Eed*-hom oocytes. Data represent the mean transcripts per million reads (TPM) for each gene from N = 5 *Eed*-wt, 4 *Eed*-het and 6 *Eed*-hom females. For classically imprinted and X-linked genes, expression levels were highly varied across some genes and have therefore been graphed for genes with low (TPM < 1), medium (TPM 1 to 5) or high (TPM > 5) expression. For all genes FDR < 0.05 in *Eed*-hom vs *Eed*-het oocytes, Error bars represent mean ± standard deviation. **Fig. S5**: Loss of *Eed* in growing oocytes did not impact expression of LINE-1 transposons. (a) Percentages of total input reads which aligned to a LINE-1 (L1) element. (b) Number of reads which map to unique and multiple L1s. (c) Proportions of reads according to the number of sites mapped to per read, to a maximum of 20. For (a-c), data represent individual replicates from *Eed*-wt (n = 5), *Eed*-wt Cre (n = 2), *Eed*-het (n = 4) and *Eed*-hom (n = 6) females. **Fig. S6**: *Eed *oocyte DEGs were not dysregulated in pre-implantation embryos. Venn Diagram comparing *Eed* oocyte DEGs against DEGs identified in *Eed* maternal null morula and blastocyst embryos. Six genes (*Plxnd1, Tceal8, Rap2c, Bbx, Xlr3c* and *Trm2b*) were common in oocytes and morula embryos, five genes (*Chrdl1, Lonrf2, Trim6, Cyp1b1* and *Ccbe1*) were common in oocytes and blastocyst embryos, and two genes (*Tspan6* and *Gk*) were common in morula and blastocyst embryos. For full DEG lists see Tables S1, S6 and S7. Morula and Blastocyst data sets were generated by analysis of published raw data sets (Manuscript References [33, 34).

**Additional file 2:** Excel file containing a Summary of all Supplementary tables and sheets containing individual Supplementary Tables.

## Data Availability

With exception of the RNA-sequencing data generated in this study, all data are available in the main text, the Supplementary Materials. The RNA-sequencing data have been deposited in the Gene Expression Omnibus (GEO) and are publicly available with accession number GSE193582. Published data sets used in this study are summarised in the Supplementary Materials. All other information is available from the corresponding author.
