# Supplementary material for: Transient Polycomb activity represses developmental genes in growing oocytes
Source: Clin Epigenetics. 2022 Dec 21;14:183. doi: 10.1186/s13148-022-01400-w (PMC9769065; doi:10.1186/s13148-022-01400-w)
Supplement: Supplementary file 1 — Additional file 1: Fig. S1: Zp3Cre-mediated Eed deletion results in loss of EED in oocytes from the primary follicle stage in Eed-hom females. Representative images of EED (red) immunofluorescence analysis in primary (top), secondary (middle) and antral (bottom) follicles in females producing Eed-wt and Eed-hom oocytes. a. shows widefield follicle images and b. shows magnified images of the area inside the white squares containing the oocyte nucleus. Lamin B1 (green) marks the nuclear lamina and delineates the edge of the oocyte nucleus in b. DAPI (blue) shows DNA in somatic cells. Images are representative of two ovaries from three biological replicates. Scale bars: 20 μm. Fig. S2: Deletion of Eed in oocytes moderately increased the rate of Surrounded Nucleolus (SN) GV oocytes compared to Non-Surrounded Nucleolus (NSN) GV oocytes. Percentage of SN (left) and NSN (right) GV oocytes obtained from Eed-wt, Eed-het, and Eed-hom females during oocyte collections. *P < 0.05, one-way ANOVA plus Tukey’s multiple comparisons test, N = 7 Eed-wt, 5 Eed-het and 7 Eed-hom females. Error bars represent mean ± standard deviation. Fig. S3: Loss of Eed in growing oocytes did not impact the transcription of genes encoding other core PRC2 subunits, PRC1 core components or DNMTs. Expression of core (a) PRC2 and (b) PRC1 components, and (c) DNMTs in Eed-wt, Eed-het and Eed-hom oocytes. Data represent the mean transcripts per million reads (TPM) for each gene from N = 5 Eed-wt, 4 Eed-het and 6 Eed-hom females. FDR < 0.05 for Eed only, error bars represent mean ± standard deviation. Fig. S4: Eed deletion results in up-regulation of a subset of imprinted and X-linked genes in Eed-hom GV oocytes. Expression of core (a) putative H3K27me3-imprinted (b) classically imprinted, and (c) X-linked Eed oocyte DEGs in Eed-het and Eed-hom oocytes. Data represent the mean transcripts per million reads (TPM) for each gene from N = 5 Eed-wt, 4 Eed-het and 6 Eed-hom females. For classically imprinted and [file 13148_2022_1400_MOESM1_ESM.pdf]

Supplementary Tables and Figures

Title: Transient Polycomb activity represses developmental genes in growing oocytes.

Authors: Ellen G. Jarred[1], Zhipeng Qu[2], Tesha Tsai[1], Ruby Oberin[1], Sigrid Petautschnig[1], Heidi Bildsoe[1], Stephen Pederson[2], Qing-hua Zhang[3], Jessica M. Stringer[3], John Carroll[3], David K. Gardner[4], Maarten van den Buuse[5], Natalie A. Sims[6], William T. Gibson[7], David L. Adelson[2], Patrick S. Western[1]*

Affiliations: [1]Centre for Reproductive Health, Hudson Institute of Medical Research and Department of Molecular and Translational Science, Monash University, Clayton, Victoria, Australia; [2]Department of Bioinformatics and Computational Genetics, School of Biological Sciences, University of Adelaide, Adelaide, South Australia, Australia; [3]Biomedicine Discovery Institute, Monash University, Clayton, Victoria, Australia; [4]School of BioSciences, University of Melbourne, Parkville, Victoria, Australia; [5]School of Psychology and Public Health, La Trobe University, Melbourne, Victoria, Australia; [6]Bone Cell Biology and Disease Unit, St. Vincent's Institute of Medical Research and Department of Medicine at St. Vincent's Hospital, Fitzroy, Victoria, Australia; [7]Department of Medical Genetics, University of British Columbia and British Columbia Children's Hospital Research Institute, Vancouver, BC, Canada.

* Corresponding author: patrick.western@hudson.org.au

References are included in the main manuscript

Please note: Supplementary Tables 1-10 are included as a separate Supplementary excel file with this submission

Supplementary Figures:

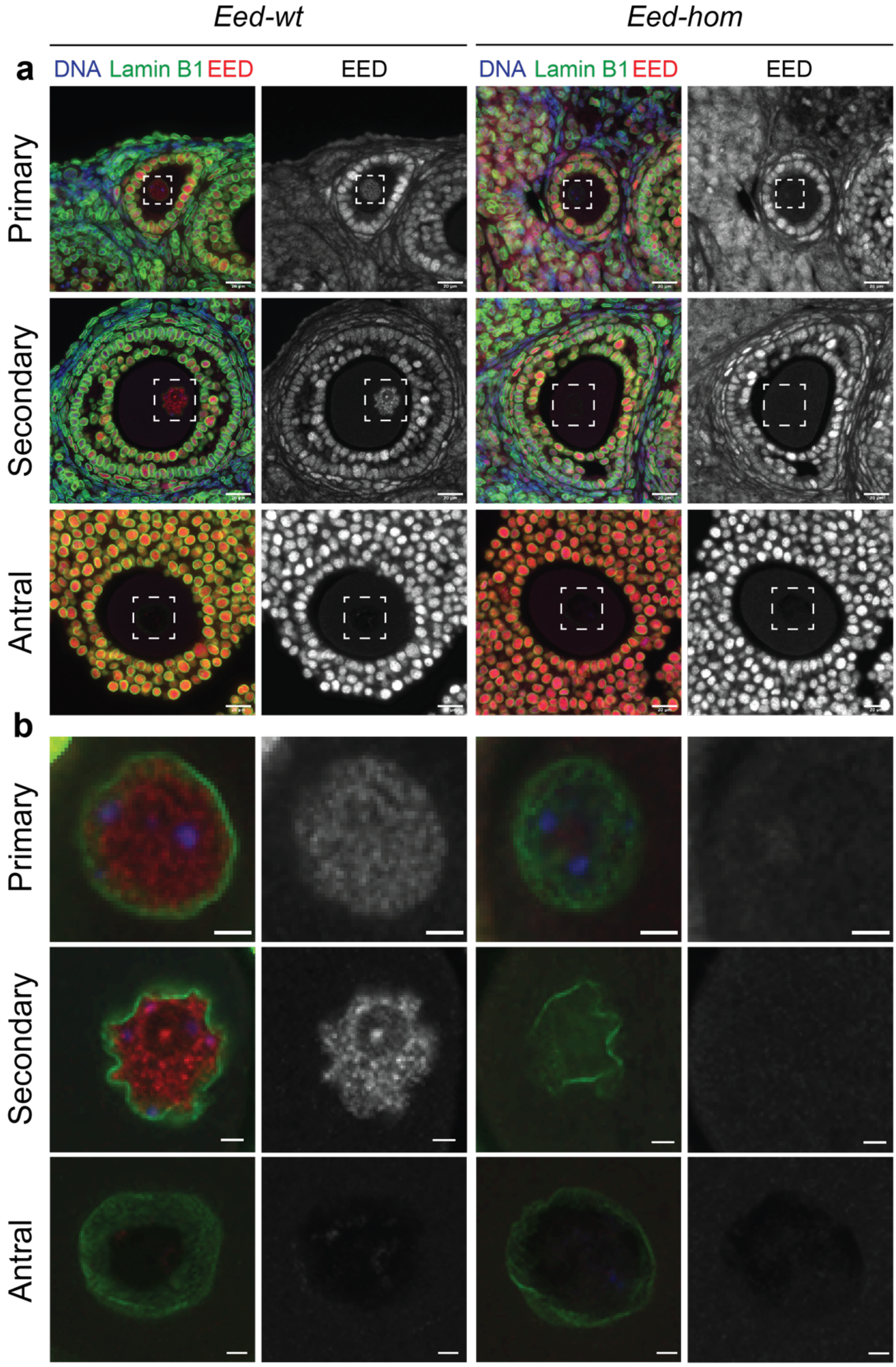

Supp. Fig. 1. ***Zp3Cre*-mediated *Eed* deletion results in loss of EED in oocytes from the primary follicle stage in *Eed-hom* females.** Representative images of EED (red) immunofluorescence analysis in primary (top), secondary (middle) and antral (bottom) follicles in females producing *Eed-wt* and *Eed-hom* oocytes. **a.** shows widefield follicle images and **b.** shows magnified images of the area inside the white squares containing the oocyte nucleus. Lamin B1 (green) marks the nuclear lamina and delineates the edge of the oocyte nucleus in **b**. DAPI (blue) shows DNA in somatic cells. Images are representative of two ovaries from three biological replicates. Scale bars: 20 µm.

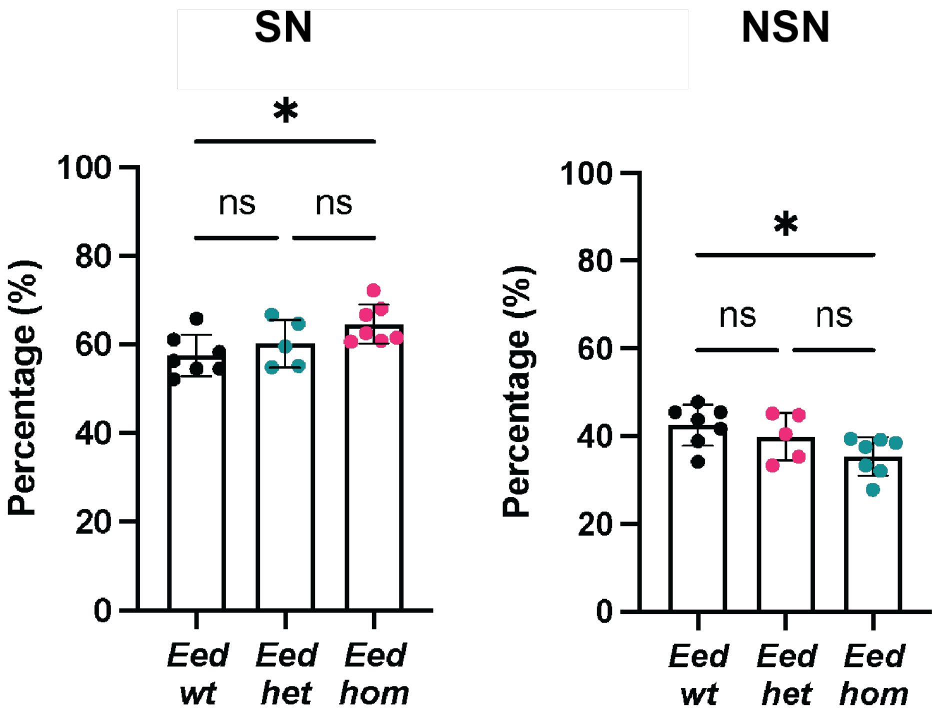

Supp. Fig. 2. Deletion of *Eed* in oocytes moderately increased the rate of Surrounded Nucleolus (SN) GV oocytes compared to Non-Surrounded Nucleolus (NSN) GV oocytes. Percentage of SN (left) and NSN (right) GV oocytes obtained from *Eed-wt, Eed-het,* and *Eed-hom* females during oocyte collections. *P < 0.05, one-way ANOVA plus Tukey's multiple comparisons test, N = 7 *Eed-wt*, 5 *Eed-het* and 7 *Eed-hom* females. Error bars represent mean ± standard deviation.

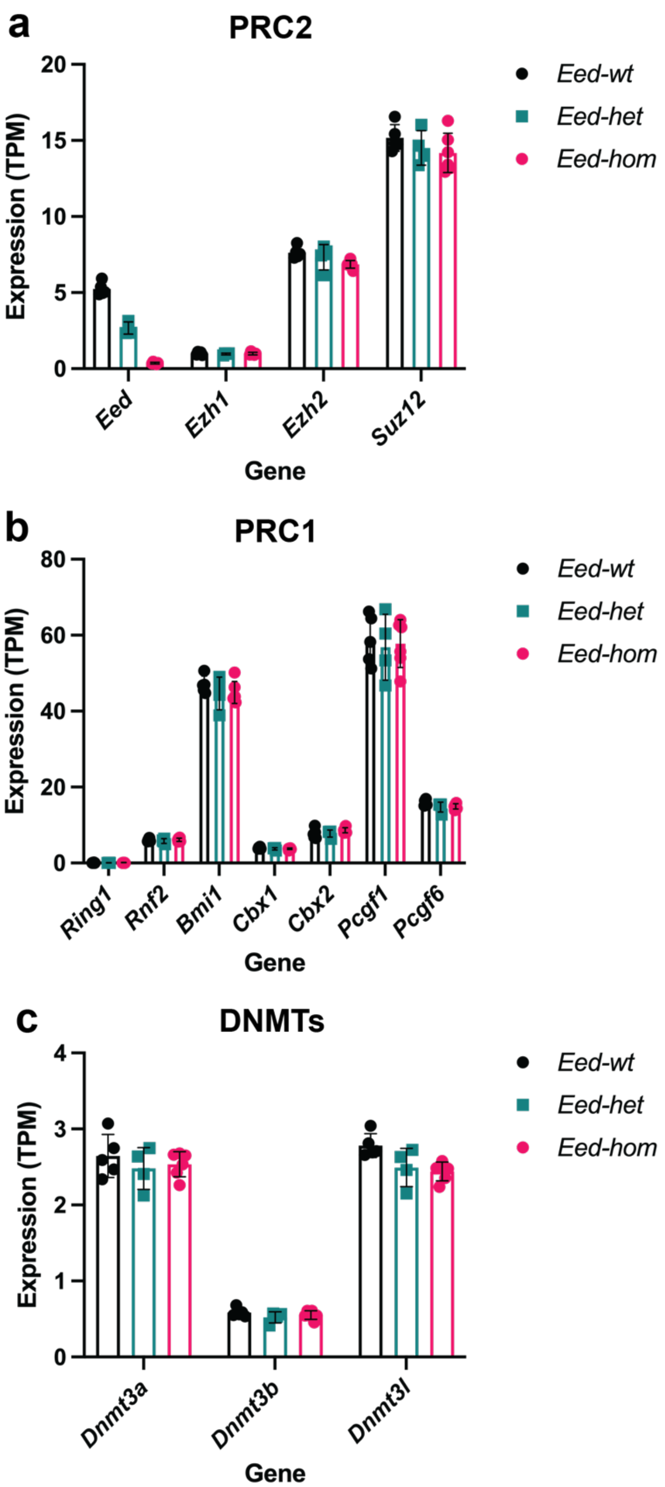

Supp. Fig. 3. Loss of *Eed* in growing oocytes did not impact transcription of genes encoding other core PRC2 subunits, PRC1 core components or DNMTs. Expression of core **(a)** PRC2 and **(b)** PRC1 components, and **(c)** DNMTs in *Eed-wt, Eed-het* and *Eed-hom* oocytes. Data represent the mean transcripts per million reads (TPM) for each gene from N = 5 *Eed-wt*, 4 *Eed-het* and 6 *Eed-hom* females. FDR < 0.05 for *Eed* only, Error bars represent mean ± standard deviation.

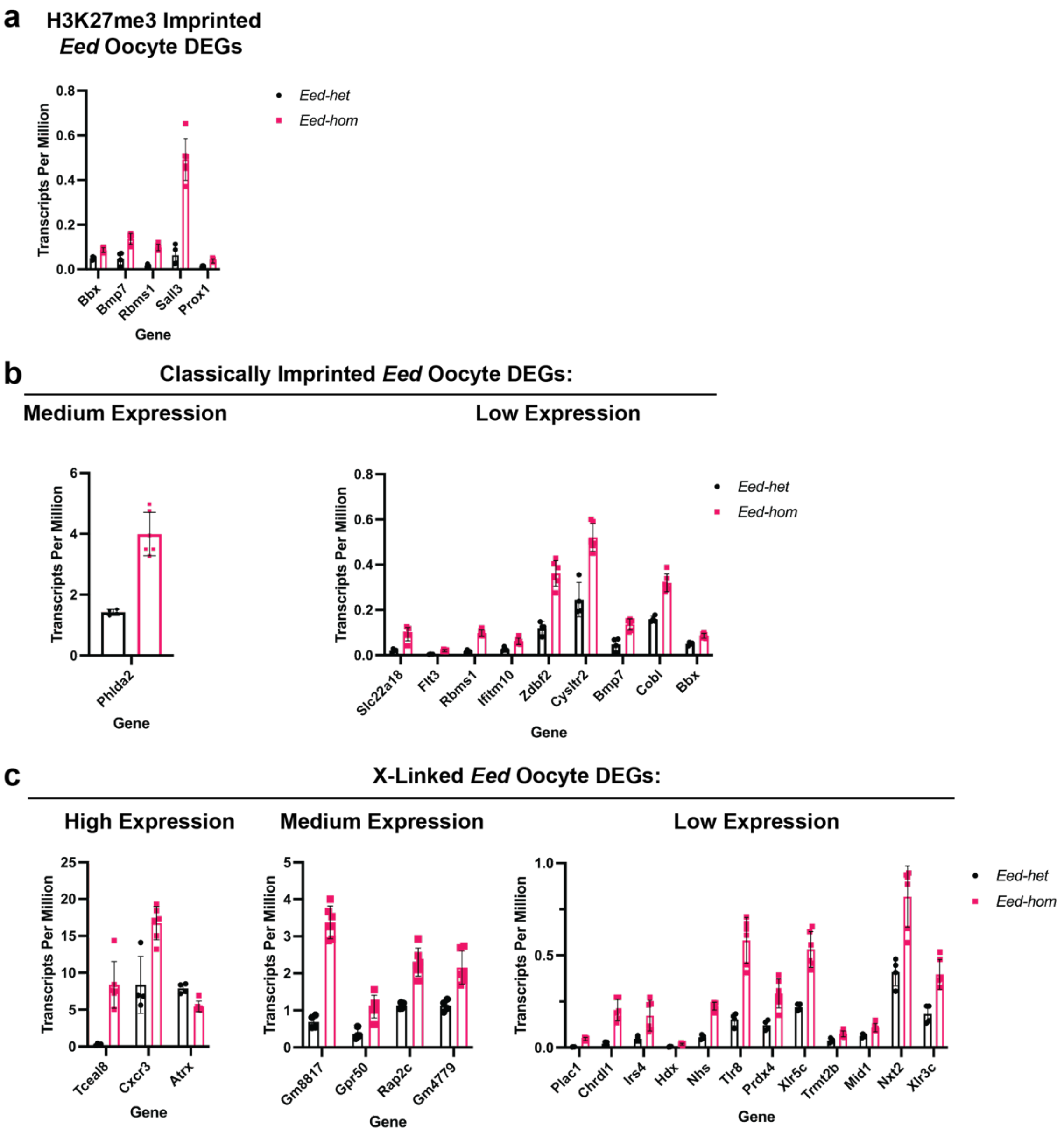

Supp. Fig. 4. *Eed* deletion results in up-regulation of a subset of imprinted and X-linked genes in *Eed-hom* GV oocytes. Expression of core **(a)** putative H3K27me3-imprinted **(b)** classically imprinted, and **(c)** X-linked *Eed* oocyte DEGs in *Eed-het* and *Eed-hom* oocytes. Data represent the mean transcripts per million reads (TPM) for each gene from N = 5 *Eed-wt*, 4 *Eed-het* and 6 *Eed-hom* females. For classically imprinted and X-linked genes, expression levels were highly varied across some genes and have therefore been graphed for genes with low (TPM < 1), medium (TPM 1 to 5) or high (TPM > 5) expression. For all genes FDR < 0.05 in *Eed-hom* vs *Eed-het* oocytes, Error bars represent mean ± standard deviation.

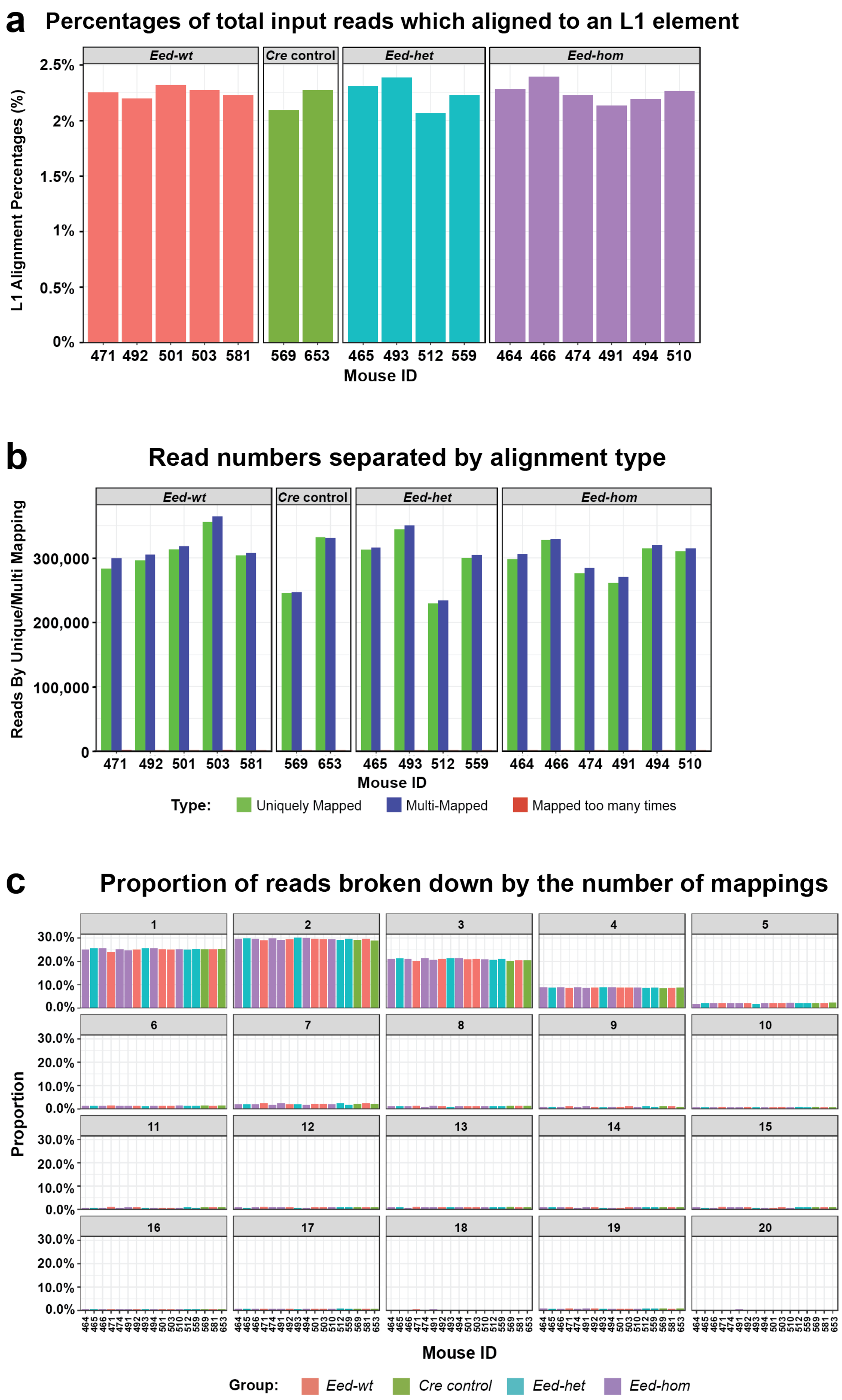

Supp. Fig. 5. Loss of *Eed* in growing oocytes did not impact expression of LINE-1 transposons. (a) Percentages of total input reads which aligned to a LINE-1 (L1) element. **(b)** Number of reads which map to unique and multiple L1s. **(c)** Proportions of reads according to the number of sites mapped to per read, to a maximum of 20. For **(a-c)**, data represent individual replicates from *Eed-wt* (n = 5), *Eed-wt Cre* (n = 2), *Eed-het* (n = 4) and *Eed-hom* (n = 6) females.

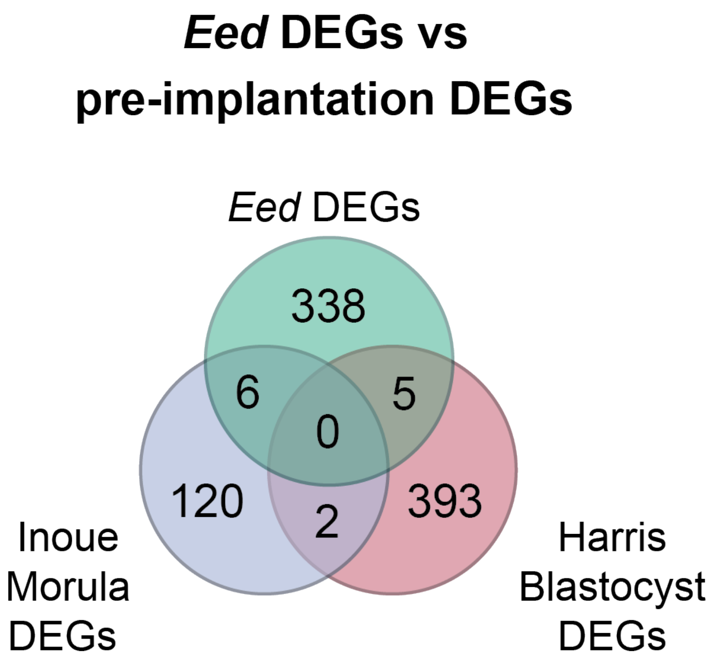

Supp. Fig. 6. *Eed* oocyte DEGs were not dysregulated in pre-implantation embryos. Venn Diagram comparing *Eed* oocyte DEGs against DEGs identified in *Eed* maternal null morula and blastocyst embryos. Six genes (*Plxnd1*, *Tceal8*, *Rap2c*, *Bbx*, *Xlr3c* and *Trm2b*) were common in oocytes and morula embryos, five genes (*Chrdl1*, *Lonrf2*, *Trim6*, *Cyp1b1* and *Ccbe1*) were common in oocytes and blastocyst embryos, and two genes (*Tspan6* and *Gk*) were common in morula and blastocyst embryos. For full DEG lists see Tables S1, S6 and S7. Morula and Blastocyst datasets were generated by analysis of published raw datasets (Manuscript References 33, 34).
